# Engaging with patients in research on knowledge translation/implementation science methods: a self study

**DOI:** 10.1186/s40900-022-00375-5

**Published:** 2022-08-08

**Authors:** Martha L. P. MacLeod, Jenny Leese, Leana Garraway, Nelly D. Oelke, Sarah Munro, Sacha Bailey, Alison M. Hoens, Sunny Loo, Ana Valdovinos, Ursula Wick, Peter Zimmer, Linda C. Li

**Affiliations:** 1 School of Nursing, University of Northern British Columbia, 3333 University Way, Prince George, BC V2N 4Z9 Canada; 2 School of Epidemiology and Public Health, University of Ottawa, Ottawa, Canada; 3 Centre for Implementation Research, The Ottawa Hospital Research Institute, Ottawa, Canada; 4 Arthritis Research Canada, Vancouver, BC Canada; 5 Health Research Institute, University of Northern British Columbia, Prince George, BC Canada; 6 School of Nursing, University of British Columbia, Okanagan, Kelowna, BC Canada; 7 Rural Coordination Centre of BC, Vancouver, BC Canada; 8 Department of Obstetrics and Gynaecology, University of British Columbia, Vancouver, BC Canada; 9 Centre for Health Evaluation and Outcome Sciences, Vancouver, BC Canada; 10 BC Centre for Ability, Vancouver, BC Canada; 11 Centre for Research on Children and Families, Montreal, QC Canada; 12 Department of Physical Therapy, University of British Columbia, Vancouver, BC Canada; 13 Patient Partner, Michael Smith Health Research BC, PaCER Certified, University of Calgary, Calgary, AB Canada; 14 University of British Columbia, Okanagan, Kelowna, BC Canada; 15 University of Northern British Columbia, Prince George, BC Canada

**Keywords:** Knowledge translation, Implementation science, Methods research, Patient and public research, Patient author, Patient-orientated research, Self-study

## Abstract

**Background:**

In 2017, the British Columbia (Canada) SUPPORT (SUpport for People and Patient-Oriented Research) Unit created six methods clusters to advance methodologies in patient and public oriented research (POR). The knowledge translation (KT)/implementation science methods cluster identified that although there was guidance about how to involve patients and public members in POR research generally, little was known about how best to involve patients and public members on teams specifically exploring POR KT/implementation science methodologies. The purpose of this self-study was to explore what it means to engage patients and the public in studies of POR methods through the reflections of members of five KT/implementation science teams.

**Methods:**

Informed by a collaborative action research approach, this quality improvement self-study focused on reflection within four KT/implementation science research teams in 2020–2021. The self-study included two rounds of individual interviews with 18 members across four teams. Qualitative data were analyzed using a thematic analysis approach followed by a structured discussion of preliminary findings with the research teams. Subsequently, through two small group discussion sessions, the patients/public members from the teams refined the findings.

**Results:**

Undertaking research on POR KT/implementation science methodologies typically requires teams to work with the uncertainty of exploratory and processual research approaches, make good matches between patients/public members and the team, work intentionally yet flexibly, and be attuned to the external context and its influences on the team. POR methodological research teams need to consider that patients/public members bring their life experiences and world views to the research project. They become researchers in their own right. Individual and team reflection allows teams to become aware of team needs, acknowledge team members’ vulnerabilities, gain greater sensitivity, and enhance communication.

**Conclusions:**

The iterative self-study process provided research team members with opportunities for reflection and new understanding. Working with patients/public team members as co-researchers opens up new ways of understanding important aspects of research methodologies, which may influence future KT/implementation science research approaches.

**Supplementary Information:**

The online version contains supplementary material available at 10.1186/s40900-022-00375-5.

## Plain English summary

In this self-study we aimed to understand how members of research teams work together. We explored how research teams included patients and public members in studying research approaches (methods) that support the creation, sharing, and use of research results that matter to patients and the public (patient and public-oriented research). We analyzed interviews with 18 members of 4 teams and discussed the findings with each team. We then further refined the results and their interpretation with patients/public team members.

- We found that research teams had to deal with the uncertainty that is common for this type of research, which is often exploratory and focuses on processes.
- There needed to be a good match between patient/public members, the topic, and the team.
- Teams had to be flexible in how they worked together and they needed to address how power was shared within the team.
- Finally, the teams were affected by world events such as COVID and social unrest.

Research team members had a chance to reflect and gain new understandings through the self-study. A key learning was that patients and public members bring their life experiences and world views to the project. They become researchers in their own right. When team members have a chance to reflect they can become more aware of their own and others’ talents, needs, and vulnerabilities, that awareness can help improve communication and teamwork. Patients and public members as co-researchers can bring new ways of understanding important features of knowledge translation and implementation science research approaches.

**Supplementary Information:**

The online version contains supplementary material available at 10.1186/s40900-022-00375-5.

## Background

Involving patients and the public in health services and clinical research is increasingly important as a factor in delivering high quality research that is meaningful to those it may impact most [1–4]. Patient-oriented research (POR) engages patients and the public in all stages of the research process [1, 2]. In POR, the term, patient, usually refers to those who live with a health issue, their families, and their caregivers [1] and the term, public, more broadly includes community members who may be affected by health and social care services or policies [2, 3, 5]. The patient and public engagement-in-research movement parallels the findings of research that engaging stakeholders in knowledge translation (KT) and implementation science research enables research outcomes to be more relevant and able to be implemented [5, 6]. While involvement of patients, the public, knowledge users, and other stakeholders is a sign of high-quality patient-oriented and integrated KT research [7], it is not commonplace to involve patients and the public in methodological research on KT/implementation science [8–10].

The knowledge, skills, attitudes, and supports that are necessary for patient engagement in research on clinical topics have been well explored. Competencies required by patients, researchers, clinicians, and policy makers in POR were identified in a scoping review by Frisch et al. [2]. The two critical elements for patients were lived experience in the condition being studied and an interest in participating in research. In addition to these two elements. Frisch et al. [2] emphasized that patients’ competencies needed to include knowledge, skills, and personal characteristics relevant to the particular project. In most studies with patients on research teams, the research has primarily relied on patients’ experience with a particular health issue [11].

Recent research usefully articulates what constitutes meaningful patient engagement [12], ethical comportment in engaging with patients in research [13], lessons learned in patient-engaged research [14], social processes that academic researchers use to involve patients in POR [4], and competencies required by researchers and patients for POR [2, 15]. Hamilton et al.’s [12] Patient Engagement in Research Framework (PEIR Framework) characterizes meaningful patient engagement in research as including convenience, team interactions, supports, and feeling valued as elements that create a positive environment for patients within a research team.

Through a relational ethics approach, Leese et al. [13] examined patients’ experiences of benefits and risks of engaging as co-researchers in partnered research. Three themes were articulated: being heard, co-building social relations, and adding another spinning plate to an already busy life. These themes illuminated how researchers and patients “ought to treat each other” when engaging with each other in research. These themes dovetail with Witteman et al.’s [14] 12 practical lessons learned about working with partners, including patients. The 12 lessons were organized in three themes: establishing and maintaining a culture of respect, actively involving all team members, and facilitating good communication. Kimminau et al. [16] usefully delineated how patient and community engagement may be similar or different, and operate with consistent underlying principles. Key principles of how effective POR and other partnered research teams work together are those of “reciprocal relationship, colearning, honesty, and trust” [17]. When patients are involved in methodological research on implementation, the roles may differ due to the particular focus of implementation research [10]. In addition to different roles, the focus of methodological research in KT/implementation science may influence how research teams develop, as well as how patients, the public, and other stakeholders are engaged. Without a direct focus on healthcare programs or services, KT/implementation science methodological research teams need to maintain their focus on methods within changing practice and policy environments where players and organizational priorities shift. Co-production of knowledge in KT/implementation science methods demands “commitment, persistence and sufficient resources” [18], p. 130].

Competencies and frameworks for POR teams are valuable and important, but do not address the temporal and processual realities of research team development and functioning that are necessary for teams to develop strengths and work through times of tension. Zibrowski et al. [4], in their realist review of patient engagement in POR, outline mechanisms, largely connected to the research context, that enable academic researchers to develop trust, cultural competence, reduce power differentials, respectful team environments, and recognize experiential knowledge, support patients to feel valued, and ensure readiness to undertake research. Salazer et al. [19] hold that research teams develop integrative capacity, that is the ability to overcome barriers created by different disciplines, knowledge, and backgrounds. POR research teams may have unique opportunities to generate integrative capacity. Engaging patients in studies of POR methodologies remains unexamined. A self-study conducted with POR teams working on KT/implementation science methodologies allowed for this exploration.

### BC SUPPORT Unit KT/Implementation Science Methods Cluster

In British Columbia, the Canadian Institutes of Health Research’s national initiative, Strategy for Patient-Oriented Research (SPOR) provincial SUPPORT (SUpport for People and Patient-Oriented Research) Unit created six methods clusters to advance methodologies in POR [20]. The BC SUPPORT Unit KT/Implementation Science Methods Cluster funded five investigator-initiated projects [21]. The teams were diverse in their composition and process of involving patients or the public, with each involving at least one patient/public member on the team. The teams studied the following methodological approaches: consensus methods used in integrated KT to promote POR, a hermeneutic approach to implementation science, the creation of online systems-thinking tools for community groups, the development of an online portal for citizen science, and using documentary as a method of KT to reach the “sandwich generation”, those who care for both their own children and ailing parents [21]. Even though all teams were led by senior researchers with extensive experience in patient/public and other stakeholder engagement, involvement of patients and the public on KT/implementation science methods-focused research teams was a new experience for some of the team leads. After consulting with their respective teams, the team leads thought that a self-study process would assist them in optimizing team functioning and meaningful engagement. The purpose of this quality improvement-focused self-study was to enhance the development of the research teams. It explored what it means to engage patients and the public in studies of POR methods through the reflections of members of the KT/implementation science teams.

## Methods

### Study design

Between 2019 and 2021, we undertook a self-study of research teams’ experiences in conducting methodological studies of KT/implementation science approaches to POR. The self-study provided opportunities for research teams to engage in collaborative reflection. Through that reflection, the teams could better understand how to engage with patients and the public as co-researchers as well as to develop the teams.

The self-study was guided by the questions:

- What does it mean for teams to include patients, the public, and other stakeholders as partners in methodological research in patient-oriented KT/implementation science?
- In what ways can research teams work through common issues encountered in methodological research in patient-oriented KT/implementation science?

### Self-study approach

Our approach to the self-study was informed by collaborative action research, which can be described as a way of thinking and acting in human inquiry [22]. We conceptualized self-study as a methodology for studying professional practice that was self-initiated, collaborative, and aimed toward improvement through learning from experience [23]. The goal was to solve problems and through critical reflection and dialogue, to understand differently, to learn and act in new ways [24]. That is, through collaborative inquiry we would extend understandings that could lead to new ways of working within the research teams as well as enhanced relationships within and across the research teams. Consistent with collaborative action research, we undertook iterative cycles of designing or planning, collecting data, analyzing data, reflecting together, communicating, and taking action [22, 25]. Identifying knowledge, principles, or theory that was useful beyond the research teams was considered a benefit and allowed us to gain insights about conducting patient-oriented KT/implementation science methodological research.

### Self-study participants: researchers and patients/public

Members of all five teams in the KT/Implementation Science Methods Cluster [21] participated in the self-study, which was led by two senior researchers, one who was the lead of the KT/Implementation Science Methods Cluster (LCL) and the other with expertise in collaborative action research (MLPM). Both researchers also led a research team in the Methods Cluster. With the addition of another research team lead (NDO) and the research associates, they became the overview team that directed the self-study planning and problem-solving. The other two research leads (AB and SM) provided input at various points. Two research associates (JL and LG), each with over 10 years of experience in qualitative research, facilitated meetings, conducted interviews with the participants, managed and analyzed the data, and prepared the preliminary interpretations and an initial publication [26]. All five teams indicated support for the self-study; however, one chose to limit its participation due to feasibility for team members to engage in the process during the self-study period. Patients/public team members were involved in interviews, discussions, interpretations, and as co-authors. As this was a self-study, we did not collect participant demographics.

### Self-study process

**Fig. 1:**
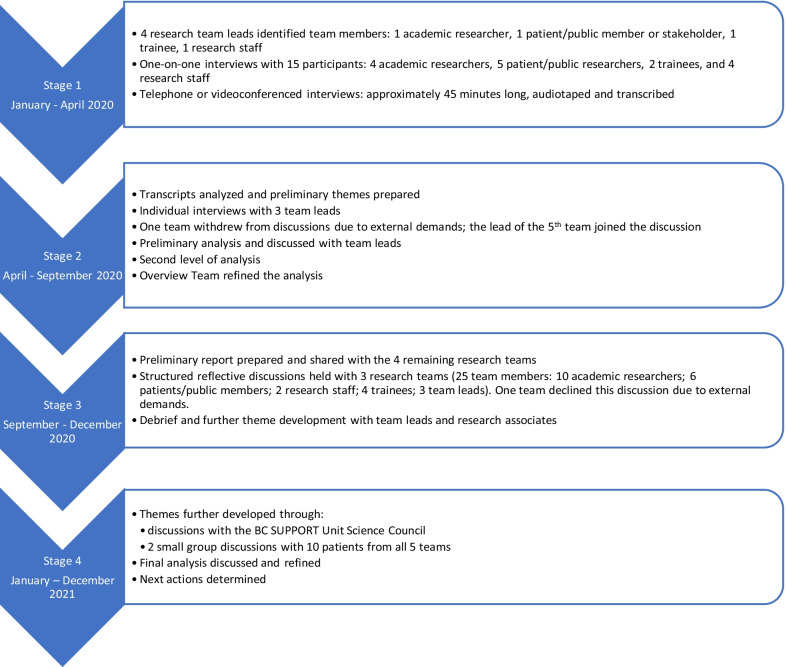
Self-study process

The self-study was carried out through a four stage, iterative process depicted in Fig. 1.

Stage 1 included individual interviews about participants’ experiences of patient/public engagement at various stages in POR within their KT/implementation science methods team. Interview questions are available in Additional file 1.

Stage 2 focused on the first level and second levels of analysis and theme development.

In Stage 3, four teams discussed the preliminary themes through structured reflective discussions around the following questions:

1. Does this report resonate with your experience?
2. How relevant is it for patients/public partners to be on KT/implementation science methods research teams?
3. What advice would you have for future research teams engaging in POR KT/implementation science methods?
4. How do you think we should communicate this and who would be involved in communicating it?

The original plan was for research teams to take the findings and implement actions, followed by another round of interviews. The teams said however, that no specific changes were needed as team leaders and research coordinators had already made adjustments to improve team functioning such as increasing the frequency and amount of communication directly with individual team members. Team members declined a second iterative cycle but emphasized the value of the structured discussion for sharing viewpoints and reflecting together as a team.

The themes were further developed through analysis of data from the facilitated discussions, combined with research team leads’ experience over the turbulent year of the COVID-19 pandemic. The team leads and research associates reflected on what it meant to lead POR KT/implementation science methodological research teams during this time [26].

In Stage 4, final themes were developed following a presentation to the BC SUPPORT Unit Science Council and small group discussions with patients/public members from all five teams. They addressed the question, “Where is there resonance and what needs to be reconsidered?” Actions consisted of communicating the self-study findings and approach with other research teams and potential team members in meetings, conferences, and publications.

### Data analysis

Data were analyzed through processes of reflexive thematic ﻿analysis [27] with thematic framework analysis [28] in Stage 2. The two research associates independently coded samples of early transcripts, line-by-line, with no pre-selected codes. They then clustered initial codes into preliminary themes and by constantly comparing the data with the themes, applied the preliminary themes to subsequent transcripts. The intent was to identify commonalities and differences among the teams and roles of team members. The second level of analysis in Stage 2 by one of the co-leads (MLPM) consisted of synthesizing the preliminary themes into three overall themes of what it means to engage patients and the public in POR KT/implementation science methodological research. In Stages 3 and 4 the research leads, with input from team members undertook further reflexive analysis and interpretation and created the final four themes.

### Trustworthiness

Extensive dialogue amongst the team members and the iterative process of the self-study contributed to trustworthiness of the findings. While coding data and creating preliminary themes, the research associates discussed differing assumptions or interpretations of the data to achieve rich interpretations of meaning. There were many discussions of the data, most importantly with patients/public members, to create a plausible interpretation, one that resonated. Through these ongoing discussions and as the analysis progressed, we continually kept our perspectives and assumptions in question.

## Findings

The self-study addressed the experiences of research teams and how the teams worked through issues they encountered in the course of POR KT/implementation science methodological research. Four themes detail the challenges experienced and how teams addressed them. The themes are undertaking KT/implementation science methodological research that explores approaches and processes, making a good match in forming a research team, maintaining well-functioning research teams through times of uncertainty and change, and being attuned to the external context and its effect on the team. Quotes are identified with pseudonyms and team member positions (Lead, Academic, Patient/Public, Trainee (graduate student), Research Staff).

### Undertaking KT/implementation science methodological research: working the process

Through the course of the self-study, we explored whether KT/implementation science methodological research was different than other forms of POR, KT, or implementation research that involved stakeholders on research teams.

Team members spoke about how they experienced methodological research approaches differently than clinically or service-focused projects. They said that it was less “concrete” than other projects. The lack of concreteness was experienced by members of four teams, “In methodological [research], you talk about how to work the process” (Eddie, Patient/Public). This focus on process was unfamiliar to many. “When we started this project, we were all dealing with something that we didn’t know” (Bev, Trainee). The focus on process meant that the projects remained dynamic and exploratory, requiring openness on the part of the team members. “Honestly I didn’t know what to expect… We’ve been figuring it out as we go” (Pat, Patient/Public). This openness demanded that team members develop trust and be flexible. As Dana (Patient/Public) said, “there really needed to be a tremendous amount of flexibility”. Even though the project direction was clear, the steps to get to the goals were created as the project unfolded. A direct relationship to participants’ health conditions, health care, or health services was non-existent or tenuous in all projects. As Bev (Trainee) said, the project is “so far away from patient care, in a sense, and so open.”

The projects differed in terms of their development. Members of the team that studied the translation of knowledge to a specific audience through familiar media did not find the method to be as vague or exploratory. Nevertheless, some patients/public members still noted the need of a clear “roadmap” in order to feel comfortable on the research team.

### Forming the research team: making a good match

Each research team approached the formation of the team differently. Members on some teams had longstanding relationships, including with patients/public members, while on other teams, most members met for the first time. In the Stage One interviews, team members talked about finding a fit with both the people and the project. A good fit was described in terms of having shared research interests, a common purpose, shared values and philosophical alignment, or achieving mutual benefit; that is, a fit with both the team and the type of research. “[There’s] a good fit with the priority problem that we’re working on ….” (Ivy, Academic).

In subsequent discussions, patients/public members identified the word, “match”, to be a better descriptor than “fit”, as “match” reflected their experience of agreeing to match their skills and knowledge to the needs and goals of the team, to be full members of the research team. Match reflected their perceptions of the importance of being on an equal footing with others on the team, rather than fitting into or being “assimilated [into a pre-arranged] research world” (Billie, Patient/Public), largely in a secondary role.

#### Creating a diverse, relevant team

The teams were diverse. As one member said, “I’m working with people in different disciplines than I would typically be working with” (Harry, Academic). Forming the teams took time and specific, intentional actions on the part of the team leads and team members to identify individuals who could fill different roles for the methodological study requirements. One patient/public member, for example, was also an expert in media technology, a needed skill on one of the teams. Another team benefitted from a patient/public member’s experience in community engagement. Clinical fit was not particularly of concern in these methodological studies.

The methodological focus meant creating new teams. “On this project I’m working with all new researchers and partners… that was because [Lead] reached out to all of us and had a conversation with us about our different priorities and then did a little bit of matchmaking” (Ivy, Academic). The research leaders drew on previous relationships and networks. For example in a community-based research project, “We had developed such strong partnerships with different organizations and so it’s never a difficulty to find partners that are patients because we’re always in contact with them, they’re always interested in what we’re doing. It’s an ongoing relationship, whether or not there’s a project going on.” (Sue, Research Staff).

Some teams experienced gaps in their composition over the course of the project, when it became evident that some voices were missing. Some patients/public members expressed the need for both representativeness and diversity. They thought that by engaging patients/public members who had previously worked together, the team would hear “the same voices over and over and over again” (Eddie, Patient/Public), the team would “not adequately reflect” (Francis, Patient/Public) the community, and the study would not benefit from “the diversity of experiences” (Eddie, Patient/Public). Some patients would also be prevented from having “a chance to participate” (Pat, Patient/Public). Other patients/public members, who spoke about the importance of research expertise gained during their time on the teams, identified alternative perspectives about engaging new patients/public members, including engaging those with team experience. They raised the point that patients/public members’ expertise may be as important as expertise amongst the researchers and that “having the relationship with the researchers [allows patients/public members to] work much better with somebody that you know, that you’ve worked with” (Billie, Patient/Public).

Matches could also be limited due to structural issues related to uneven power dynamics, such as daytime research meetings, that were set to accommodate academic members’ schedules, when some patients/public members who worked could not attend. Compensation for work time missed or expenses were other potential barriers to engagement.

There’s a structural thing, in terms of barriers. We’ve designed a system that works well for the researchers. And then we end up with a specific group of people that can fit into that time. Then we get the same old people over and over again. And then we say, “Oh, there’s no diversity.” (Billie, Patient/Public)

Matches were made when team members recognized connecting agendas, came to common understandings, and appreciated differing perspectives. A good match occurred when team members gained and maintained a sense of shared purpose and mutual benefit. Not surprisingly, academic researchers and patients/public members came to the project with different and sometimes overlapping expectations and perspectives. Both came to the project with, “the expectation of learning more about the situation …. and how to influence change” (Eddie, Patient/Public) and consensus about “… what they want the outcome of the study to be” (Alex, Patient/Public). Differences in involvement lay in “**why** [academics and patients/public members] want that outcome to come to fruition” (Alex, Patient/Public), or how the process of engaging with the team could benefit themselves. Some noted differing reasons for involvement. Chris (Patient/Public) noted that goals were different, that: “…goal as academics is the research project” and what they can gain out of it, while patients/public members became engaged as part of their own care and healing, or with the question of how “it's going to affect me, personally going forward? (Eddie, Patient/Public).

#### Moving from a good match to a functioning team

Initial discussions between team leaders and team members, often before the team’s first meeting, were critical to ascertain and affirm the various members’ perspectives and agendas in an unfamiliar area of research. As Laird (Academic) said, “it was to try and find what people’s thinking around this approach was and what their experiences were. And they’re all different”. One patient/public member recalled a meeting over lunch with an academic that allowed them to get to know each other, to gain some common understandings, and to clarify their own and each other’s perspectives. These initial discussions were helpful in allowing team members to hear and trust each other.

The small connections that were already there were very, very helpful… it was easier to get more acquainted and to be more open to – everybody needs to approach this openly, being ready both to listen and to speak. And to have people around you that you recognize a little bit. Just the slightest connection, it will give a platform for further discussions. (Jules, Trainee)

Becoming a functioning team took time and specific, intentional actions on the part of the team leads and members. Taking time to get to know fellow team members and roles is commonplace at the start of a research project. Becoming a functional team, however, was complicated by the methodological focus on process, the interconnections with the social, political, and/or healthcare contexts that are part of KT/implementation science research, as well as the exploratory approach taken by some teams.

Methodological research’s lack of concreteness sometimes exacerbated feelings of vulnerability, especially for the patients/public members and trainees, who indicated a lack of confidence, particularly when a language barrier prevented them from understanding the many technical components of the discussions, or hampered their confidence in speaking out. The lack of a specific, clearly delineated content topic was challenging for some members and may have affected how they contributed. More clarity meant “what is the purpose… what is the end goal…what is expected of the patient… we need a more definitive road map” (Chris, Patient/Public). Greater clarity was needed by some to feel that their participation was not “a waste of time….” (Kim, Patient/Public). Despite the desire for clarity, a definitive road map at the project’s outset was not possible for most of these methodological research projects.

One patient/public member suggested that the match with the research team be treated as a volunteer opportunity that could be enhanced by an interview and a clear job description of what was expected of the patient/public member, and what they specifically would get in return. The match was also about the team being ready for the individual patient/public member, “to mentor, to bring them along” (Francis, Patient/Public). Most of the team leads spoke of actively developing their teams throughout the project, at the same time as completing the research.

### Maintaining well-functioning research teams: working intentionally yet flexibly

The teams found that they needed to work flexibility, yet intentionally as they implemented their projects. Throughout the projects, the teams needed to actively work to maintain ongoing agreement on a common purpose, keep a patient/public focus on methodological issues, and explicitly attend to communication, power dynamics, and language. How the team leads and research coordinators enacted their roles was key in these efforts.

#### Keeping the focus on patient/public oriented methodological research

Even though the initial team development steps focused on sharing perspectives and coming to a common understanding, teams needed to regularly communicate and revisit the goals and understandings throughout the project.

Communication to gain understanding of each other and the goal needs to be happening all the time. Consistently going through checkpoint milestones, throughout the course of the study. You can’t expect to sit down in a meeting, talk about it, come to an understanding and then that's it, and expect [that understanding to be the same] throughout the course of a one to two year project. (Eddie, Patient/Public)

Several of the patients/public members talked about how easy it was to “lose the plot” (Chris, Patient/Public), that is, lose track of the steps of the research, not only because of the gaps between meetings, but also because of the vagueness of the research, the focus on process, and the unfamiliarity with much of the methodological language. As Chris (Patient/Public) said, “It is easier to lose the plot as it is not directly relevant to what is important in your everyday life.” The experience of losing the plot also contributed to a lessening of some patients’ confidence to contribute to the discussions.

Patients/public members played a number of roles in the teams, drawing on the skills and perspectives they brought in from their life experiences, occupations, and skills. One important action was to ground the teams that were often focused on exploring concepts and process.

And he says, “as a patient it is this…” When he speaks, I can see very clearly that that’s the angle he’s taking at this meeting. To me that’s very helpful. I think it’s been very helpful for the group. It’s kept us grounded. (Rae, Team Lead)

They were also essential in keeping the teams on track to implement the methodological research plans in a patient-focused manner. For example, “At each stage [patient/public member] would be reminding us to be mindful of what is the patient voice in this or how does the patient need to be considered” (Sue, Research Staff).

Patients/public members made important contributions, not only in ensuring clarity and relevance, but also supporting the expression of vulnerability and supporting humility within the team.

And [patient] was very vocal when it came to, I don’t understand what you are talking about. What does this mean? … I think it had an effect on the atmosphere of the group. It was allowing everybody to show their limitations, in a sense. He helped develop that atmosphere of being secure in the group [to ask questions] because that helps everybody. (Bev, Trainee)

#### Actively leading the team: team leads and research coordinators

The team leads played pivotal roles in developing the teams and keeping them functioning through approaches that were intentional, respectful, and sensitive. When team leads actively incorporated patients/public members as researchers within the team, they felt supported, connected, and on equal footing within the project, regardless of societal roles. As Rae (Lead) said, “That’s what I wanted to establish. As not a professional role or patient role but as people on a team”. This was accomplished, from the patients/public members’ perspective, when the team leads recognized the vulnerability of the members, and portrayed “an attitude, like total respect, totally listening to what I was saying” (Chris, Patient/Public). This approach in the “little moments” (Kim, Patient/Public) helped to “give weight to the moments” without condescension. It went “a long way to building that self-esteem and relationship with the team” (Chris, Patient/Public).

The team leads’ skills were critical in helping the patients/public members to be heard. “I would say, there’s nobody in this group that… doesn’t have their voice heard…. Part of it is having an academic lead who is mindful of that… and lots of experience facilitating team meetings” (Mel, Academic). A trainee noted the effect of working in this way. “It creates the atmosphere of respect, of being listened to and listen to others and make sure that everybody has a voice” (Jules, Trainee).

Maintaining connections and fostering ongoing, productive actions took a lot of effort and time on the part of the team lead and the team’s research coordinator, including conversations outside of regular team meetings. As Laurie (Lead) said, “There is a significant amount of time that you need to set aside to build relationships with your patient partners and to have that review of communication outside of the team meetings with them”. This process clarified conversations, gave patients/public members, who might not be as forthcoming in the larger group, time to voice their concerns, and allowed teams to be flexible in implementing their research plans. As Nora (Academic) observed, “[Lead] has had to have additional conversations about, okay, we need to be flexible with this or that, because it now looks different”. This sensitive attention to communication also provided the opportunity for some team members, who continued to feel lost well into the project, to find the plot again.

#### Working through differences in perspectives, power, and language

Team members found working with differing perspectives to be a messy process, often repetitive, and frustrating. A common purpose helped, but the teams did not find a straight-forward path to achieving it. The process reality of methodological research required flexibility, achieved through extended discussions, respecting other’s views, listening for understanding, and trusting. The process took time. “Just having those conversations about what people understood things to be, and then trying to find that middle ground, of kind of collective agreement of what we were actually trying to do here.” (Harry, Academic).

Patients/public members noted the importance of acknowledging the power imbalance. “At the end of the day. I don't have the final say. And it's the researcher who has more power than me” (Billie, Patient/Public). One team consciously incorporated the principle of reciprocity, with time built into each meeting for personal updates and sharing news. The Lead always scheduled patients/public members to speak first and checked in with them by telephone before making major decisions so they could take a team-based approach and deflect potential power imbalances.

Some patients/public members wanted to be part of each and every step of the research process, “I'm really struggling with even the concept of not being included in every team meeting, when I'm part of a research project, because things are said and done” (Francis, Patient/Public). Others were satisfied with various levels and types of engagement at different stages of the research.

Finding a common language was not straightforward. “We have to be really mindful about the language we’re using to make sure that it’s inclusive and not intimidating for folks… it is an ongoing struggle” (Jan, Patient/Public). The language of “patient partners” was contested by the patients/public members as it denoted an assistant role rather than a full researcher role. One suggestion was that in POR, the language be “patient researchers and non-patient researchers” (Alex, Patient/Public). “I know that I'm making a difference, just the same as any non-patient researcher can know that they're making a difference” (Alex, Patient/Public).

To address the dynamics of methodological research and the issues that arose during the research, teams needed flexibility to make changes to the project when in progress. “It’s a bit of a dance to get it right” (Gerry, Lead). Humility, flexibility, and responsiveness to patients/public members’ concerns were especially needed when external issues influenced the studies’ implementation and approach.

### Being attuned to the external context and its influence on the team

The teams continued their work throughout the pandemic and the societal shifts that happened during that time, including the Black Lives Matter movement and the Canadian commitments to Truth and Reconciliation with Indigenous Peoples. These events required the teams and team leads to reflect on handling change, building relationships in the teams and enabling different perspectives to be heard [26].

When events outside the teams’ control interrupted the research project, such as COVID 19, teams initially halted work. “Everything stopped because of COVID… everything shut down” (Rae, Lead). They regrouped and continued with other activities (e.g., preparation of documents) and reworked their plans to respond sensitively to the external context, through addressing the process of the research.

[COVID] essentially halted the project. We were in the process of developing and finalizing a development stage…. And that’s essentially not going to happen at this point in time at all. And I don’t know when it will… We’re trying to move on phase 2 and phase 3 … together so that they’re happening iteratively. (Laurie, Lead)

One project’s focus and content changed to incorporate the reality of the pandemic’s impact on health service delivery, “everything has changed…” (Rae, Lead). Another team, “has had to have additional conversations about, okay, we need to be flexible with this or that, because it now looks different…” (Harry, Academic). In this time of anxiety and uncertainty, several teams expressly talked about how to deal with changes, "Because it is an empathy and a respect and a genuine care for everyone on the team” (Diana, Academic).

The Black Lives Matter movement intensified awareness of issues of membership, equity and racism in the context of methodology and the team. There was a call to expand who was involved and a suggestion on one team that patients/public members should not work with the team more than once. Teams also reconsidered knowledge translation products and their accessibility to different groups. They raised the importance of breaking usual assumptions and ways of working. “We need to get out of way, break the cycle of’same old, same old patient’. We have to change and do better for people out there in the community who are not privileged” (Francis Patient/Public).

Addressing these issues in the midst of already non-concrete, processual approaches to methodological research was challenging. The team leaders and research coordinators were key in ensuring ongoing communication and helping the groups to keep focused on the research so as not to “lose the plot”, while surfacing the tensions and working through them, all through the electronic communications required by the pandemic.

## Discussion

This self-study of teams undertaking POR in KT/implementation science methodologies addressed two issues. One was what it means for methodological research teams to include patients/public and other stakeholders and the other was to explore how research teams can work through common issues encountered in this type of research.

### Doing patient-oriented methodological research

The self-study revealed how the patients informed the methodological approaches and how the teams accomplished the research. The patients/public’s voice infused the ways in which the methodological approaches were conceptualized and carried out, and in turn, influenced what we learned about doing methodological research. We learned that the patients/public’s concern of “nothing about us without us” was not only important in studies of implementing services or programs [29], but also important in advancing KT/implementation science methods.

It is not intuitive, nor always easy, to fully involve patients/public in teams studying KT/implementation science methodological research, with its exploratory nature and focus on process rather than on a substantive patients/public-related concern. The self-study discussions illustrated that patients/public members brought their life experiences, including their occupational knowledge and skills, and their world views to the teams, not only their experience with a condition, group, or location [4]. As a result, patients/public members raised new conceptualizations, new issues, and provided needed expertise as well as unique perspectives to the teams. Their professional and work experience/knowledge, as well as their lived experience contributed to the accuracy, validity, and completeness of the studies. These contributions were not always anticipated prior to the study. The team leads and other group members, through their thoughtful, intentional actions, were key in helping the patients/public members’ knowledge, expertise, and voices be heard and incorporated into the research [2, 4, 14].

The metaphor of a *dance over time* was apt given the need for the team lead and research coordinator to provide supportive, graceful repetition of steps and adding new steps over time. As the COVID pandemic led to changes in meeting and communication formats, along with delays or revisions to research methods, the team leaders had to reorient team members several times. It took time for team members to come to repeated common understandings about the project, the steps to be taken, and members’ roles. Throughout these processes patients/public members always focused on how to address patient/public member needs within the method under study. In these methodological projects, it took more effort and much longer to get to a place of common understanding, coordination, and smooth action. While responsive, repetitive, supportive actions on the part of POR research team leads are implicit within the POR literature [2, 4, 12], they become explicit when addressing the process requirements of these KT/implementation science methodological studies.

The teams had to handle change and uncertainty, maintain a sense of shared purpose and mutual benefit over time, sustain relationships and relationship-building, and foster diversity in ways of involving patients/public members. While these actions are not unique to POR methods teams [2, 4, 12, 14], the time-intensive and processual nature of the KT/implementation science methodological studies and the turbulent external situations exacerbated the demands on the teams and amplified issues of power, vulnerability, and power imbalances.

#### Addressing power within the team

POR often highlights the importance of equitable power, the realities of power imbalances in research teams, and the need to explicitly attend to imbalances to enhance patient engagement and research outcomes [4, 30–32]. Principles of reducing power differentials are part of patient engagement best practices [31]. Less examined however, is the nature of power and vulnerability within teams [33]. POR in KT/implementation science, with its lack of certainty, focus on conceptualization, and lack of direct relevance to patients/public members, may bring issues of power and vulnerability to the forefront.

In our self-study, some patients/public members said they wanted to be included in all team meetings and in all research stages as a way to equalize power with academic researchers. Others disagreed and raised the point that there are other ways of understanding equitable power by differentiating between structural power and moral power. In POR teams, academic researchers hold structural power by virtue of their place in the team and accountability for funding, project implementation, and completion. POR methodological research, however, requires full patient/public involvement and as a result, patients/public members hold considerable moral power. Moral power is “the degree to which an actor, by virtue of his or her perceived moral stature, is able to persuade others to adopt a particular belief or take a particular course of action” [34]. The patients/public members’ moral power was demonstrated in many ways: patients/public members kept discussions grounded, determined some implementation steps, identified gaps in knowledge translation approaches, and questioned inclusiveness in team membership and research approaches. They also brought forward the ways that different patients/public members’ voices and perspectives were heard and encouraged or overlooked.

With the exception of noting the vulnerability of patients/public members, vulnerability within research groups is seldom directly addressed [33]. In our KT/implementation research teams, patients/public, trainees, and researchers all expressed feelings of vulnerability, of feeling uncertain, open to emotional exposure, or risk [35]. The vulnerability of trainees and patients/public members, sometimes expressed through not speaking up, stemmed from sometimes being lower in structural power. Team leads, who were often short on moral power, experienced vulnerability when team members expressed concern about lack of inclusiveness in project design. When the team lead and team members did not take a defensive stance, through actions such as expressing a lack of knowledge, maintaining respectful language, keeping discussions open, and being open to new ways of understanding project design, vulnerability was accepted as a strength, rather than a weakness [36]. Power relationships can be balanced when teams respect patients’ moral power and incorporate it into respectful deliberations and ongoing action.

Language and action are key in bridging gaps in power among team members. One insight was that naming patients/public members of teams as partners puts them in a secondary role to academics, the researchers. A suggestion was to say that patient researchers and non-patient researchers comprise POR teams. Some patient/public team members were insistent that they not be named ‘patients’ as they were either on teams studying implementation of health promotion interventions to the healthy public or had conditions where naming them as patients could be stigmatizing.

In POR about particular health topics, it may be easier to conceptualize patient/public researchers as those who have lived experience with a health condition(s), and other researchers as those who do not have the lived experience. However, in POR in KT/implementation science, where patients/public team members do not need to have experience with a particular health condition or situation, it is difficult to think of who would be the non-patient researchers. All team members are patients at some point and are members of communities and the public. Differences in roles perhaps relate to research as an occupation, which is often the case for academics, but may not be as marked, even for those highly engaged patients/public members who may be considered to be “professional patients” on research teams [37]. Discussions led the teams to conclude that patients/public team members fully contribute to KT/implementation science methodological research through their life experiences and world views.

### Engaging in a self-study

Even though involving patients/public is expected to enhance the focus and outcomes of research [4, 37], we were not sure what involvement of patients/public in methodological research would actually be like. The original intent of the self-study was to assist the teams to develop by providing the opportunity for them to engage in collaborative reflection on ways of working through common issues. An unexpected benefit was how the self-study allowed for a new perspective on KT/implementation science methodological research itself. Individual and group opportunities for reflection provided new understandings, especially as the methodological focus of the studies was not familiar to most team members.

We also gained insights on how engaging in self-study revealed taken-for-granted aspects of team development and team functioning. Structured reflection proved to be a catalyst, helping the teams to recognize previously underappreciated scope and depth of issues [33]. It helped to expose strengths of team members as well as commonalities in experiences within and across different teams and team members.

Individual and group reflections contributed to enhanced awareness of team needs, greater sensitivity, and communication within teams. Reflections highlighted how continuing attention is needed for successful group functioning. The deliberative discussions helped team leads and research coordinators to remain attuned to team members’ issues and concerns, particularly in the context of external social, political, and/or healthcare forces. Several team leads took action, adjusting their approaches to meet the research goals while better supporting team members. For example, teams adjusted meeting timing, sequencing of research activities, and approaches to data collection and analysis to address changes in the healthcare system due to COVID and the needs of the patient/public members.

Research team members appreciated the chance to be interviewed and provide feedback. Specific meetings with patients/public members created new emphases in the self-study findings, as some patient/public voices were heard for the first time during the self-study. Some patients/public members said that the separate meetings in the self-study allowed them to be more confident in expressing their thoughts than in the meeting of their whole team. While reviews indicate that providing opportunities for patients’ voices to be heard is important [31, 38], only a few detail how and when those opportunities can best be provided [13].

Without the final separate sessions with the patients/public members in the self-study, when they were asked about resonance of the findings and what was missing, the meaningfulness of power dynamics and how they played out in language may have been overlooked. The team leads were aware of power as an ongoing issue but its emphasis in the findings was revised to include the realities for patients/public members. POR researcher competencies reflect the need to be finely attuned to group needs and group development, while explicitly attending to power differentials [2], but how to address these issues is seldom part of researcher training.

The iterative reflection of this self-study illuminated the folly of pigeon-holing patients/public members in POR as being representative of a perspective [39]. Patients/public members added an essential grounding for all team members and provided contributions that broadened the understandings of KT/implementation science methodologies and self-studies. Through the lens of reinterpretation, the self-study allowed for reflection upon “moments of disruption in our practice” [40, p. 276]. The processes led to new learning and action both individually and collectively.

See Table 1, considerations for a self-study of a patient/public-oriented research team studying KT/implementation science methods, and the infographic: how to do a self-study of a patient/public oriented research team in Additional file 2.

**Table 1:** Considerations for a self-study of a patient/public-oriented research team studying KT/implementation science methods

**Why should a research team undertake a self-study?**
To explore members’ individual and collective experience
To engage in individual and collective reflection
To address a goal of quality improvement with the ability to gain knowledge, principles or theory
**When to undertake a self-study?**
Readiness to engage with patients and the public as co-researchers
Readiness to have enhanced awareness of team needs and greater sensitivity and communication among team members
Readiness to explore how the team can work through common, potentially challenging issues and willingness to act on the findings and insights
Ability to adjust team approaches to meet the KT/implementation science methods research goals while supporting team members
**Which approach?**
Collaborative action research, or another approach that allows for iterative processes of exploration and self-reflection
**What process?**
The process is collaboratively determined and may include:
Individual interviews
Analysis of interviews and identification of findings that may be themed
Reflective discussion of findings with research team
Team decisions about action to take about any findings or insights
Analysis of team discussion and/or potential interviews
Further theme development through joint reflection with team members
Action with team members and others on themes and overall findings
**How to implement a self-study?**
Start conversations and planning for the self-study early among team members, with sensitivity to power relationships within research teams
Involve team members in decision-making about the self-study, including the implementation/interpretation of data, discussions of reporting and deciding who have access to the data
Clarify ethical issues; tools such as the A Project Ethics Community Consensus Initiative Screening Tool (https://arecci.albertainnovates.ca) can be useful
Engage with team members with qualitative methodological expertise
Involve interviewers from outside of the team to conduct interviews and directed reflections to allow for openness and anonymity/ confidentiality
Consider specific needs of patient/public members re interviews and/or separate reflective discussions
Consider who should see raw data or only themed, anonymized data
Create opportunities for discussions among the team that may bring out new understandings
Consider that action may happen in the near or distant future. Action may take the form of activities, developing new skills, using and sharing new concepts and/or influencing change. Action may also be a conscious team decision to not take specific actions, but to work with greater understanding

### Limitations

A variety of perspectives on the challenges were experienced within the research groups, including tensions that happened and how the group moved through these tensions. Some individuals experienced challenges more sharply than others. Not all teams participated throughout the entire self-study process due to external constraints. We may not have fully captured the diverse experiences of members of the research teams. At the same time, a diverse mix of research team leaders and members participated from across all of the KT/implementation science methods cluster research teams. As only two trainees participated in this study, the trainee experiences may warrant further exploration. The initial interviews were analyzed by the two research associates for confidentiality purposes. As patients/public members did not analyze the raw data, some important features of patient/public involvement may have been missed. As well, interviews were not conducted by patients/public members, which may have limited the patient/public voice.

## Conclusions

This self-study has allowed members of research teams to reflect on and articulate how patients are engaged in furthering KT/implementation science POR methods. Our reflections contribute to the growing literature that can support meaningful practices with POR to adapt and persist in turbulent times of fast-moving change. Perhaps differently than other forms of POR, KT/implementation science methodological research needs to attend to the social, political, and/or healthcare contexts in which knowledge is being translated or implemented.

The involvement of patients in KT/implementation science methodological research illuminates the exploratory nature of this type of research. Patient-oriented KT/implementation science methodological research places extraordinary demands on the research team leader and research coordinator due to the uncertainty of the research trajectory, its exploratory approach, and its interconnections with external contexts. One demand is to engage all team members for what they can contribute given their expertise and background, and not just their roles. The second demand is for exquisite communication and group facilitation skills, to keep the patients/public members, in particular, from “losing the plot”, assuring consistent equality of power among all members of the team, and maintaining everyone’s full engagement. These demands have illuminated how methodological research amplifies the responsibilities and skills required of team leaders, as well as the time required for research, even more so than for other types of POR. Finally, the double reflection of the self-study provides a heightened awareness that patients/public members on research teams come with full biographies and all types of experiences. It is through these full biographies that they contribute their invaluable knowledge and play critically important roles on methodological research teams.

## Supplementary Information

**Additional file 1.** Interview questions.

**Additional file 2.** Infographic: how to do a self-study of a patient/public oriented research team.

## Data Availability

The data generated or analysed during the self-study are not publicly available due to confidentiality commitments to participants.

## Abbreviations

BC SUPPORT Unit: British Columbia SUpport for People and Patient-Oriented Research Unit
KT: Knowledge translation
POR: Patient oriented research
