# Supplementary material for: Engaging with patients in research on knowledge translation/implementation science methods: a self study
Source: Res Involv Engagem. 2022 Aug 8;8:41. doi: 10.1186/s40900-022-00375-5 (PMC9358643; doi:10.1186/s40900-022-00375-5)
Supplement: Supplementary file 1 — Additional file 1. Interview questions. [file 40900_2022_375_MOESM1_ESM.docx]

**Engaging with Patients in Research on Knowledge Translation/Implementation Science Methods: A Self Study**

**Additional File 1**

STAGE 1: INDIVIDUAL INTERVIEW QUESTIONS

- What may have contributed to any hesitancy to engage in a self-study? How should we address any concerns now, and as the process unfolds?
- Could you tell me about how the research team came to be formed?
  - How and when were the patient(s) identified/selected (for patient/other knowledge user partners – were you engaged)?
- Has there been any particular training of research project team members? How and when has that occurred? Can you say when you noticed that training came in handy?
- Would you tell me about a time (or situation) that would show what it is like for a patient to be (OR – to be a patient/knowledge user/community member [use appropriate term] on this research team?
  - What made it easy/hard?
- How has your experience of being on the team changed over time?
  - What sparked the changes – what happened afterwards?
- In what ways have the patients (have you as a patient?) contributed to the development and implementation of the project?
  - What has been easy? What has been difficult?
- How have the team and its members built on the easy parts and worked through what has been difficult?
  - What worked and what didn’t work?
- What tensions (conflicts) have been experienced? How were they addressed or worked through?
- What have you learned from working with this team/on this project? Can you give an example?
- What logistics have been in place to support your contribution/the team? Were they timely?
- If there is one thing you would want us to know about what is important about working in teams that are studying patient-oriented knowledge translation or implementation science, what would it be?
- What has worked well; what has not worked well?
- What surprises have you experienced in working with the team as you’re doing this research?
- What advice would you have for other teams working on patient-oriented KT/IS methods

STAGE 3: INTERVIEW QUESTIONS FOR STRUCTURED DISCUSSIONS WITH TEAMS

1. Does this report resonate with your experience?
2. How relevant is it for patient and public partners to be on KT IS methods research teams?
3. What advice would you have for future research teams engaging in POR KT IS methods?
4. How do you think we should communicate this and who would be involved in communicating it?

STAGE 4: QUESTIONS FOR FOLLOW UP GROUP INTERVIEWS WITH PATIENTS

1. Where is there resonance with this analysis and interpretation?
2. What needs to be reconsidered?
