# Supplementary figures and images for: Engaging with patients in research on knowledge translation/implementation science methods: a self study
Source: Res Involv Engagem. 2022 Aug 8;8:41. doi: 10.1186/s40900-022-00375-5 (PMC9358643; doi:10.1186/s40900-022-00375-5)

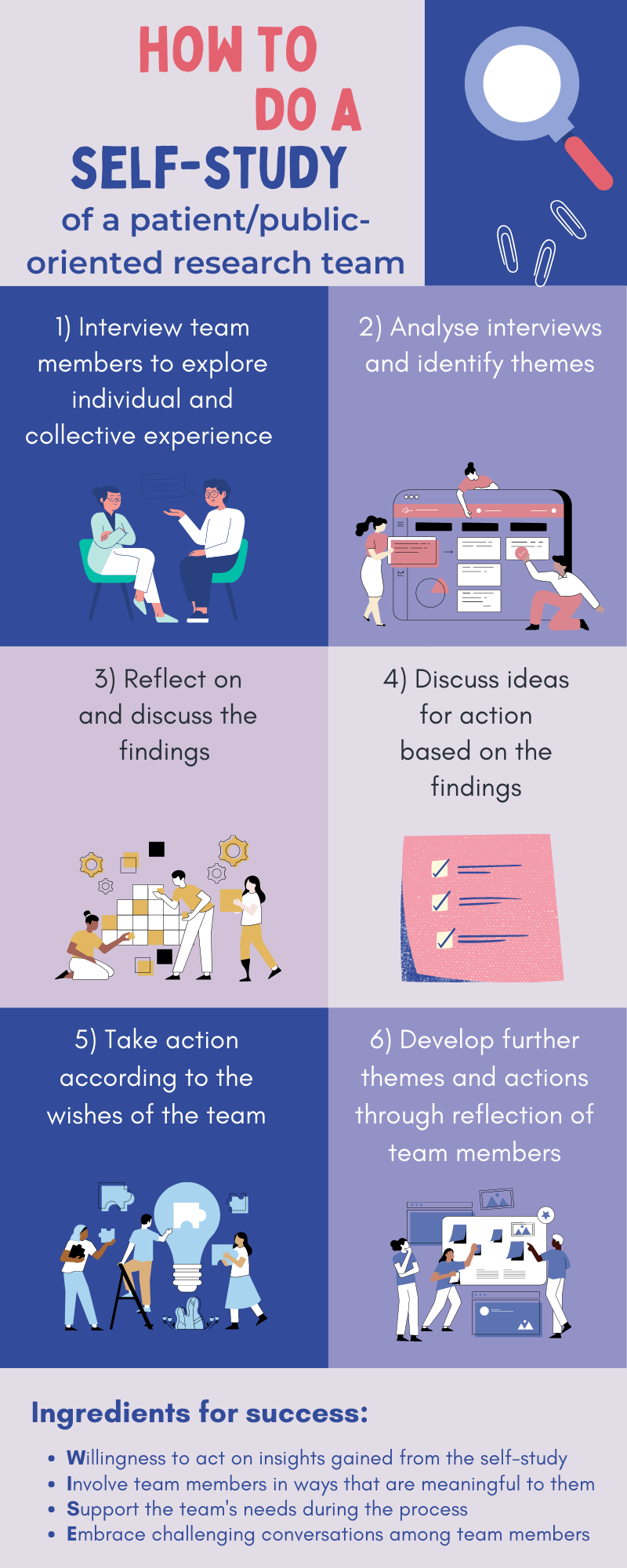

Supplement: Supplementary file 2 — Additional file 2. Infographic: how to do a self-study of a patient/public oriented research team. [file 40900_2022_375_MOESM2_ESM.png]
